# Alcohol use disorders and associated factors among people living with HIV who are attending services in south west Ethiopia

**DOI:** 10.1186/1756-0500-7-828

**Published:** 2014-11-24

**Authors:** Matiwos Soboka, Markos Tesfaye, Garumma Tolu Feyissa, Charlotte Hanlon

**Affiliations:** Department of Psychiatry, College of Public Health and Medical Sciences, Jimma University, Jimma, Ethiopia; Department of Health Education and Behavioral Science, College of Public Health and Medical Sciences, Jimma University, Jimma, Ethiopia; Department of Psychiatry, School of Medicine, College of Health Sciences, Addis Ababa University, Addis Ababa, Ethiopia; Centre for Global Mental Health, Health Services and Population Research Department, King’s College London, London, UK

**Keywords:** Alcohol use disorders, Hazardous alcohol use, Harmful alcohol use, Alcohol dependence, HIV, Sub-Saharan Africa, Mental health, Depression

## Abstract

**Background:**

Alcohol use disorders (AUDs) in persons living with human immunodeficiency virus (PLHIV) in high-income countries have been associated with poor adherence to antiretroviral medications and worse HIV-related outcomes. Little is known about AUDs among people attending HIV services in sub-Saharan Africa.

**Methods:**

Across-sectional study was carried out among PLHIV who attended HIV services at Jimma University Specialized Hospital in September 2012. The World Health Organization’s Alcohol Use Disorders Identification Tool (AUDIT) was used to measure probable hazardous, harmful and dependent use of alcohol (‘alcohol use disorders’). Associations between AUDs and other variables were explored using logistic regression analysis. All variables associated with AUDs with a p value <0.25 were included in the final multivariable model.

**Results:**

The overall prevalence of AUDs was 32.6%, with hazardous use, harmful use and alcohol dependence accounting for 24.7%, 2.8% and 5.1% of the total, respectively. There was no significant difference in the prevalence of AUDs in persons receiving antiretroviral treatment compared to those who were antiretroviral therapy naïve (32.6% vs. 38.6%). AUDs were identified in 26.0% and 44.1% of females and males, respectively. Male gender, smoking cigarettes and psychological distress were positively associated independently with AUDs.

**Conclusion:**

The high prevalence of AUDs detected in our facility-based survey of PLHIV in Ethiopia highlights the need to integrate delivery of effective and feasible interventions for AUDs into HIV care.

## Background

Alcohol use disorders (AUDs) contribute to 3.8% of the overall global burden of disease [1, 2]. The number of deaths attributed to alcohol worldwide is greater than the combined number of deaths from human immunodeficiency virus (HIV), acquired immunodeficiency syndrome (AIDS), violence and tuberculosis [3]. AUDs can be categorized as harmful use, hazardous use or dependence [4, 5]. Harmful alcohol use is characterized by an alcohol consumption pattern that is within the individual’s control but which is causing physical or mental problems and may also have social consequences [4, 5]. Hazardous use of alcohol considers both the increased risk of harmful physical, mental or social consequences for the user and harm to others. Dependent alcohol drinking is characterized by alcohol use that takes over a person’s life to the extent that they have a physiological or psychological need to continue drinking. Dependence is the most severe end of the AUD spectrum and is invariably associated with many social, psychological and physical health problems [1, 4, 5].

The prevalence of alcohol use disorders appears to be high among people living with HIV (PLHIV) compared to the general population [6]. AUDs are associated with premature mortality in PLHIV [7–9], thought to occur due to alcohol enhancing the toxicity of antiretroviral treatment (ART), increasing liver damage from concurrent infection with hepatitis C virus, and increasing the risk of opportunistic infection due to decreased effectiveness of ART and exacerbation of immune suppression [10–15]. Hazardous levels of alcohol use have also been associated with non-adherence to ART medication [3, 16–19], late presentation to HIV/AIDS care [20], higher viral load during the course of HIV treatment [15, 18], accelerated decline of CD4 cells in HIV infected persons, faster disease progression and development of resistant strains of HIV [3, 15–18]. Furthermore, alcohol use has been associated with increased HIV risk behaviors, including unprotected sex, multiple sexual partners and high-risk injection behaviors [9, 21–23]. In the USA (Veterans Aging Cohort Study), any alcohol consumption was shown to decrease overall survival in PLHIV by more than two years if the frequency of consumption was once per week or greater, and by 6.4 years with daily consumption [24]. Other studies have shown that most PLHIV fear the interaction between their medication and alcohol and, therefore, skip medication while drinking alcohol [16, 25]. Research suggests that alcohol consumption accelerates HIV/AIDS disease progression even if medications are taken correctly, by adversely impacting drug absorption and metabolism [25].

In HIV services in African hospital settings, the prevalence of AUDs has been found to vary from 12% in a teaching hospital in Nigeria [26], 15% in a Ugandan university teaching hospital [27], and 39.4% in a specialist hospital in Nigeria [28]. Factors found to be associated with AUDs in these settings include male gender, psychological morbidity, smoking cigarettes, Christian religion, lower education, peer pressure, parental modeling and drinking in order to assist social interactions [26, 28–32].

In Ethiopia, people who self-report drinking alcohol most of the time are more likely to default from ART and present late to HIV/AIDS care [20, 33]. An association between alcohol consumption and unprotected sexual behavior in Ethiopian commercial sex workers and school-age youth has also been reported [34, 35]. However, there is no information about the magnitude of specific AUDs and the associated factors in PLHIV who are in contact with HIV services in Ethiopia [36]. Such information is important in order to identify PLHIV who are at greatest risk of AUDs and to inform the development of interventions and services to meet the specific needs of people in each category of AUDs. The objective of this study was, therefore, to assess the prevalence of hazardous, harmful and dependent AUDs, and the associated factors, in PLHIV attending HIV services in Ethiopia.

## Methods

### Setting

This study was conducted in Jimma University Specialized Hospital (‘Jimma hospital’) which is located in the south western part of Ethiopia. Jimma hospital is one of the oldest hospitals in the country and provides services for approximately 9,000 inpatient and 80,000 outpatient attendances per year, serving a catchment population of about 15 million people [37, 38]. Out-patient treatment services for HIV were established in 2005 and now provide care for a total of 6,561patients: 3,524 receiving ART and 3,037 under pre-ART follow-up. In this study, data were collected from adult people living with HIV attending the Jimma hospital clinic for follow up care during September 2012.

### Study design

A facility-based, cross-sectional survey.

### Sample size assumptions and sampling procedure

The sample size was determined by assuming a prevalence of alcohol use disorders of 50%, with any particular outcome to be with 5% margin of error and 95% confidence interval of certainty (alpha =0.05). Based on this assumption, the actual sample size for the study was computed using a one-sample population proportion formula. The total sample size was 401.

### Sampling procedure

All eligible adult attendees of the ART clinic at JUSH during the study period were invited consecutively to participate in the study.

1. Outcome: Alcohol Use Disorders
  A structured questionnaire, the WHO’s Alcohol Use Disorder Identification Test (AUDIT) was used to measure AUDs [4]. The AUDIT has been evaluated over a period of two decades, and provides an accurate measure of risk of AUDs across gender, age and cultures. A multi-country validation of AUDIT in people attending primary health care in Norway, Australia, Kenya, Bulgaria, Mexico and the United States of America found that, at a cut-off score of eight or more, the sensitivity and specificity of AUDIT for AUDs were 0.90 and 0.80, respectively [4]. For this study, the AUDIT was translated into both Amharic and Afan Oromo languages, the main languages spoken in the study site. Back-translation into English was undertaken for both languages and the final versions obtained through expert consensus. The cultural appropriateness of the AUDIT was improved by modifying the ‘standard drink’, referred to in questions two and three, so as to be more understandable to participants. Local alcohol drinks, for example ‘arake’ (distilled from fermented barley or maize combined with ‘gesho’[Rhamnusprinioides]), ‘tela’ (a beer-like drink fermented from barley and Rhamnusprinioides) and ‘teji’ (honey wine) were first converted from local measurements to milliliters based on previous studies in Ethiopia that investigated the ethanol content of local beverages [39]. Then the measured alcohol was converted into a standard drink by calculating the mass and volume of the alcohol. Similarly, local beer (bottles and glasses) and wine were converted to standard drinks based on their alcohol content. Since Ethiopia has no alcohol policy defining standard alcohol drinks, the South African standard drink (containing 12 g of alcohol) was used [40]. Locally, different receptacles, including ‘malekia’, ‘tassa’ and ‘birille’ are used when drinking Arake, Tella and Teji respectively. Therefore, we measured the volume of each of these receptacles and converted the drinks to milliliters in order to obtain a standard drink that is comparable internationally.
  In accordance with the recommended scoring, a total AUDIT score of eight or more was used to define probable ‘alcohol use disorder’ (AUD). A total AUDIT score of 1–7 indicated social drinking, a score of 8–15 indicated “hazardous drinking” a score of 16-19indicated “harmful drinking” and a score of 20 or above indicated probable alcohol dependence. Harmful drinking and alcohol dependence are both indicative of severe AUD. The total AUDIT score, alcohol consumption level, signs of dependence, and markers of present harm should all play a role in determining how to manage a patient with AUD [4].
2. Potential explanatory variables for AUDs
  The following variables were assessed:
  *Socio-demographic variables:* age, gender, educational level, marital status, living conditions, ethnicity, religion and frequency of attending a place of worship
  *Socioeconomic status*: employment status, yearly income, and social support
  *Illness-specific characteristics:* WHO stage of HIV status classification and CD4 count. Self-report questions were used to assess whether the patient took ART regularly or not.
  *Mental distress:* The Kessler 6-item scale (Kessler 6) was used to measure mental distress (depressive, anxiety and somatic symptoms) [41]. The Kessler 6 has been adapted and validated in Ethiopia previously, demonstrated to have a sensitivity and specificity of 84.2% and 82.7%, respectively, at a cut-off point of 4/5 to screen for symptoms of common mental disorders [42].
  *Impaired functioning:* The 12-item WHO Disability Assessment Schedule (WHODAS) was used to assess functional impairment [43].
  *Other factors:* family history of alcohol use disorders (AUDs), cigarette smoking and use of khat (amphetamine-like psychostimulant).
3. Reasons for initiation of drinking alcohol: people who reported drinking alcohol were asked about the main reasons for starting drinking. The following response categories were recorded: easy availability of alcohol, parental modeling, peer pressure to drink, lack of somebody to provide support, drinking to increase self-confidence, drinking to alleviate fear of socializing, drinking to forget financial difficulties, longstanding life stressors, longstanding marital disharmony and family history of alcohol use disorders.

### Data collection procedures

Data were collected by seven health professionals with nursing qualifications (four of degree level and three with diploma) after three days of training to familiarize them with the instruments. Data collection was carried out after the questionnaires were pretested on a sample of (5% of the total sample) people living with HIV attending the ART clinic at Agaro health center which is 45 km away from Jimma town. The data collection was supervised by a degree level health officer. The supervisor monitored data quality and checked all questionnaires for completeness. Incomplete and unclear questionnaires were returned to the data collectors for correction.

### Data analysis

After double data entry, data were exported from EpiData and analyzed using the Statistical Package for Social Science (SPSS, version 16). Data were then cleaned. The outcome and explanatory variables were entered into a bivariate logistic regression analysis, one at a time, in order to estimate the strength of association using Odds Ratios (OR). All variables associated with AUDs in the bivariate logistic regression with a p-value less than 0.25 were entered together into a multivariate logistic regression in order to control for potential confounders.

### Ethical considerations

Ethical clearance was obtained from the Ethical Review Board of Jimma University. Written informed consent was obtained from each of the participants prior to participation. Information obtained was kept confidential and anonymous during all stages of the study.

## Results

### Participant characteristics

A total of 401 PLHIV were approached for enrolment in the study. Of these, 389 (97.0%) agreed to participate and 12 (3.0%) refused (seven females and five males).

The majority (63.2%; n = 246) of participants were female. The mean age of participants was 35.5 (SD 9.78) years, ranging from 18 to 70 years. The largest proportion of participants was aged between 25 and 34 years, accounting for 44.7% (n = 174) of the sample, followed by the 35 to 44 years age group which accounted for 30.0% (n = 117) of participants. Only 5.9% (n = 23) of participants were aged 18–24 or older than 54 years. Out of the total participants involved in this study, 85.3% (n = 332 participants) had been on ART (See Table 1).

**Table 1:** **Socio-demographic characteristics of people living with HIV attending services at Jimma University Specialized Hospital in September 2012 (n = 389)**

Characteristics		Frequency (%)
Sex	Male	143 (36.8)
	Female	246 (63.2)
Age (years)	18-24	23 (5.9)
	25-34	174 (44.7)
	35-44	117 (30.1)
	45-54	52 (13.4)
	55 and above	23 (5.9)
Ethnicity	Oromo	182 (46.8)
	Amhara	105 (27.0)
	Gurage	10 (2.6)
	Kefa	30 (7.7)
	Yem	17 (4.4)
	Others	45 (11.6)
Religious affiliation	Orthodox Christian	200 (51.4)
	Muslim	125 (32.1)
	Protestant Christian	58 (14.9)
	Catholic	6 (1.5)
Educational level	Illiterate	71 (18.3)
	Read and write only	11 (2.8)
	Primary (grades 1–8)	172 (44.2)
	Secondary (grades 9–12)	102 (26.2)
	Tertiary (grades 12 or more)	33 (8.5)
Marital status	Single	48 (12.3)
	Married	193 (49.6)
	Divorced	92 (23.7)
	Separated	15 (3.9)
	Widowed	41 (10.5)
Occupation	Unemployed	97 (24.9)
	Daily laborer	99 (25.4)
	Employed	55 (14.1)
	Farmer	9 (2.3)
	Merchant	63 (16.2)
	Student	3 (0.8)
	Retired	11 (2.8)
	Other	52 (13.4)
Living circumstances	Living alone	84 (21.6)
	Living with close family(father, mother, brother, sister, husband/wife, children)	279 (71.7)
	Living with other relatives (aunt, uncle, cousins, nieces or nephew)	22 (5.7)
	Living with non-relatives	4 (1.0)^4^

^4^Other ethnicity = Dawuro, Wolayita and Tigray, Other occupation = House wife, house servant and driver.

### Prevalence of alcohol use disorders

Nearly two thirds of participants (64%; n = 249) reported that they had drunk alcohol at some point in their life. Out of the total participants, 31.4% (n = 122) were identified as social drinkers (AUDIT score 1–7). The overall prevalence of alcohol use disorder (defined as an AUDIT score ≥8) was 32.6% (n = 127). Hazardous drinking, harmful drinking and alcohol dependence were found in 24.7% (n = 96), 2.8% (n = 11) and 5.1% (n = 20) of the participants, respectively. The internal consistency of AUDIT was within the accepted range (Cronbach’s α = 0.79).

Amongst patients who were taking ART, 31.6% (n = 105) were identified as having an alcohol use disorder. Of these, 24.7% (n = 82) were hazardous drinkers, 2.7% (n = 9) were harmful drinkers and 4.2% (n = 14) had probable alcohol dependence. The prevalence of AUDs in persons not on ART was 38.6% (n = 22).

Mental distress, as defined by a score of five or greater on Kessler 6, was found in 45.2% (n = 176) of the total sample. In patients with mental distress, 40.3% (n = 71) had an AUD, with 26.7% (n = 47), 4.0% (n = 7) and 9.7% (n = 17) having hazardous drinking, harmful drinking and alcohol dependence, respectively.

Amongst patients who missed doses of ART medication, 44.0% (n = 11) were identified as having an alcohol use disorder: 28.0% (n = 7) with hazardous use, 8.0% with harmful use (n = 2) and 8.0% (n = 2) with probable alcohol dependence. Amongst patients categorized as being in WHO’s stage two of HIV/AIDS classification, 40.6% (n = 52) had alcohol use disorders, with 30.8% (n = 53) in people with stage one and 26.6% (n = 17) WHO’s stage three. Only 21.1% (n = 4) of patients classified as stage four HIV/AIDS classification were found to have alcohol use disorders. AUD was more prevalent among patients with CD4 ≥ 200 cells/mm^3^compared to patients with CD4 < 200 cells/mm^3^ (36.7% vs. 10.3%). In patients with AUDs, 30.8% (n = 12) were functionally impaired for more than 15 days in the preceding month.

### Reasons given for initiation of alcohol use

In participants who were categorized as having AUDs, 41.7% (n = 53) said that they started drinking alcohol due to peer pressure, 33.9% (n = 43) initiating drinking because of easy availability of alcohol and 29.1% (n = 37) started to drink alcohol due to parental modeling. Among participants with AUDs, 29.1% (n = 37) reported a family history of AUDs (See Table 2).

**Table 2:** **Environmental and psychological factors associated with initiation of alcohol use disorders among people living with HIV attending services at Jimma University Specialized hospital**

Variables	N (%)
Easily availability of alcohol	43(33.9)
Parental modeling	37(29.1)
Peer pressure to drink	53(41.7)
Lack of somebody who provide support	14(11)
To increase self confidence	11(8.7)
To alleviate fear of socializing	8(6.3)
To forget financial difficulties	11(8.7)
Due to long-standing life stressors	8(6.3)
Due to long-standing marital disharmony	6(4.7)
Like the way alcohol makes me feel happy	29(22.8)
Family history of AUDs	37(29.1)

Generally, 34.8% (n = 39) of participants with AUDs preferred bottled beer, followed by the home brewed beer ‘tella’ (30.4%, n = 34), teji (honey wine) 15.2% (n = 17) and arake 4.5% (n = 5).

### Factors associated with AUDs

AUDs were present in 26% (n = 64) of females and 44.1% (n = 63) of males (crude odds ratio (COR) 2.24, 95% CI = 1.45 to 3.46). Males were more often identified as hazardous drinkers compared to females (30.1% (n = 43) vs. 21.5% (n = 53), p = 0.001). Males were also more likely to be dependent on alcohol than females (9.1% (n = 13) vs. 2.8% (n = 7), p = 0.001).

Orthodox Christians had two times increased odds of having AUDs compared to Muslims (COR = 1.99, 95% CI = 1.20-3.29) (see Table 3). Patients who chewed khat daily had more than four times increased odds of AUD compared to those patients who chewed khat weekly (cOR 4.25, 95% CI = 1.56, 11.58)(see Table 4). Patients with CD4 count of lower than 200 cells/mm^3^ had 80% lower odds of AUDs (cOR 0.20, 95% CI = 0.08-0.48).

**Table 3:** **Socio-demographic factors associated with alcohol use disorders (AUDs) among people living with HIV attending services at Jimma University specialized hospital (n = 389)**

Variables		AUDs	No AUD	P-value	Crude odds ratio	95% CI
		N (%)	N (%)			
Gender	Male	63 (44.1)	80 (55.9)	<0.001	2.24	1.45-3.46
	Female	64 (26.0)	182 (74.0)		Reference	
Age (years)	18-24	9 (39.1)	14 (60.9)		Reference	
	25-34	54 (31.0)	120 (69.0)	0.436	0.70	0.29-1.72
	35-44	43 (36.8)	74 (63.2)	0.829	0.90	0.36-2.26
	45-54	13 (25)	39 (75.0)	0.219	0.52	0.18-1.47
	55 and above	8 (34.8)	15 (65.2)	0.760	0.83	0.25-2.75
Ethnicity	Oromo	46 (25.3)	136 (74.7)		Reference	
	Amhara	37 (35.2)	68 (64.8)	0.074	1.61	0.96-2.71
	Gurage/Keffa/Yem/Others	44 (43.1)	58 (56.9)	0.002	2.24	1.34-3.75
Religion	Orthodox/Catholic	78 (37.9)	128 (62.1)	0.006	2.02.	1.22-3.33
	Muslim	29 (23.2)	96 (76.8)		Reference	
	Protestant	20 (34.5)	38 (65.5)	0.111	1.74	0.88-3.45
Frequency of attending worship	Daily/2–3 weekly	48 (28.6)	120 (71.4)		Reference	
	Weekly	67 (35.3)	123 (64.7)	0.177	1.36	0.87-2.13
	Less than a week	8 (38.1)	13 (61.9)	0.370	1.54	0.60-3.95
	Never	4 (40.0)	6 (60.0)	0.444	1.67	0.45-6.17
Education	No formal education	28 (34.15)	54 (65.85)		Reference	
	Primary	63 (36.6)	109 (63.4)	0.700	1.12	0.64-1.94
	Secondary/Tertiary	36 (26.7)	99 (73.3)	0.24	0.70	0.39-1.27
Occupation	Unemployed	30 (30.9)	67 (69.1)	0.	Reference	
	Daily laborer	29 (29.3)	70 (70.7)	0.80	0.93	0.50-1.70
	Employed/Farmer	21 (32.8)	43 (67.2)	0.80	1.09	0.56-2.15
	Merchant	20 (31.7)	43 (68.3)	0.91	1.04	0.53-2.17
	Others	27 (40.9)	39 (59.1)	0.19	1.55	0.81-2.97
Marital status	Single	17 (35.4)	31 (64.6)	0.715	1.13	0.58-2.20
	Married	63 (32.6)	130 (67.4)		Reference	
	Separated/Divorced /Widowed	47 (31.8)	101 (68.2)	0.862	0.96	0.61-1.52
Living condition	Alone	26 (31.0)	58 (69.0)	0.729	0.91	0.54-1.54
	With family	92 (33.0)	187 (67.0)		Reference	
	Other	9 (34.6)	17 (65.4)	0.865	1.07	0.46-2.50^5^

^5^Other occupation = House wife, house servant, students, retired and driver.

Other living condition = living with relative, Living with non-relative person.

**Table 4:** **Association of mental distress and use of other substances with AUDs among people living with HIV attending services at Jimma University specialized hospital ART clinic 2012 (n = 389)**

Variables		AUDs	No AUD	P value	Crude odds ratio	95% CI
		N (%)	N (%)			
Smoking cigarettes	Yes	23 (63.9)	13 (36.1)	<0.001	4.24	2.07-8.68
	No	104 (29.5)	249 (70.5)		Reference	
Chewing khat	Yes	42 (45.2)	51 (54.8)	0.003	2.04	1.27-3.30
	No	85 (28.7)	211 (71.3)		Reference	
Mental distress	Yes	71 (40.3)	105 (59.7)	0.003	1.90	1.24-2.91
	No	56 (26.3)	157 (73.7)		Reference	

There was no association between AUDs and cannabis use, living conditions, frequency of attending a place of worship, educational status, occupation, functional impairment, marital status, missing ART medication and WHO HIV/AIDS clinical stages (See Tables 3 and 4).

After adjusting for potential confounders using multiple logistic regression, it was found that the odds of having AUDs among male participants was more than two times higher when compared to that of female participants (adjusted OR (AOR) 2.23, 95% CI = 1.30, 3.83). Being in the 45 to 54 years age group was negatively associated with AUDs compared to 18–24 years age group (AOR 0.30, 95% CI = 0.09, 0.99). Being Orthodox Christian was positively associated with AUDs when compared to Islam followers (AOR 2.29, 95% CI = 1.22, 4.31). Chewing khat was not associated with AUDs in the final model. The odds of having an AUD among smokers was more than three times higher when compared to non-smokers (AOR 3.40, 95% CI = 1.38, 8.40) in the final model. The odds of having an AUD among mentally distressed patients was more than two times higher compared to those patients free of mental distress (AOR 2.24, 95% CI = 1.40, 3.64) (See Table 5).

**Table 5:** **Multivariate logistic regression of factors associated independently with AUDs among people living with HIV attending services at Jimma University specialized hospital (n = 389)**

Variables		P-value	Adjusted OR	95% CI
Ethnicity	Oromo		Reference	
	Amhara	0.54	1.21	0.66-2.24
	Gurage/Keffa/Yem/Others	0.01	2.18	1.19-4.00
Sex	Male	0.004	2.23	1.30-3.83
	Female		Reference	
Age	18-24		Reference	
	25-34	0.34	0.61	0.22-1.69
	35-44	0.38	0.62	0.22-1.77
	45-54	0.05	0.30	0.09-0.99
	55 and above	0.39	0.56	0.15-2.13
Religion	Orthodox/catholic	0.01	2.29	1.22-4.31
	Muslim		Reference	
	Protestant	0.06	2.31	1.23-4.34
Frequency of attending worship place	Daily/2-3 weekly		Reference	
	Weekly	0.06	1.62	0.99-2.67
	Less than weekly	0.74	1.20	0.42-3.41
	Never	0.31	2.14	0.49-9.38
Mental distress	Yes	0.001	2.24	1.40-3.64
	No		Reference	
Smoking cigarettes	Yes	0.008	3.40	1.38-8.40
	No		Reference	
Khat chewing	Yes	0.12	1.63	0.88-3.01
	No		Reference	

## Discussion

In this cross-sectional survey of alcohol use disorders in persons with HIV, nearly one third had a probable AUD. The overall prevalence of AUDs (32.6%), defined by an AUDIT score ≥8,was substantially higher than that found in community-based studies carried out in Addis Ababa and Butajira, Ethiopia (3.7% and 2.7%, respectively) [29, 30]. This may be due to the difference between tools used in this study (AUDIT vs. CAGE), as the CAGE is designed to detect more severe AUDs (alcohol dependence rather than harmful or hazardous drinking). Also, the prevalence of AUDs would be expected to be lower in community based studies. However, the prevalence of AUDs found in this study was similar to estimates from a case control study of factors associated with late presentation to HIV/AIDS care among PLHIV in South Wollo Hospital, Ethiopia, (33.8%) and a previous study from Jimma Hospital, Ethiopia, on defaulters from antiretroviral treatment among PLHIV (36.5%) [20, 33], although neither of these studies used a standardized questionnaire to define AUDs. Other studies from Africa also show a similar prevalence of AUDs in PLHIV. In Nigeria the prevalence of AUDs in PLHIV was 39.4% [28].

### Prevalence of AUDs

The prevalence of alcohol dependence found in our study was higher than the result from a review of substance use in Ethiopia (1.5%) [36]. This is in keeping with other studies indicating higher levels of AUDs in PLHIV compared to the general population [6]. The prevalence was less than the report from a study done on sexual workers living with HIV in India (46%) [44]. The reason for this difference may be due to the difference in the tools used to screen AUDs.

In this study, harmful alcohol use was found in 2.8% of the total participants, which differs substantially from a similar study carried out in Nigeria in which the prevalence was 28.8% [28]. In the Nigeria study, a lower AUDIT cut-off point than the recommended cut-off was employed [4].

In this study, hazardous alcohol drinking was found in 24.7% of participants. This is higher than the prevalence of hazardous drinking found in similar studies from Uganda (15.4%) [27] and Nigeria (10.6%) [28], as well as from the review of substance use in Ethiopia (3.0%) [36].

### Socio-demographic factors associated with AUDs

Alcohol use disorders among patients 25–34 age group (31.0%) were less prevalent than in the same age group in the Nigeria study (46%) [28]. Additionally, this study found AUDs to be higher among single participants than among married participants which was in agreement with the report from Nigerian study done on similar group. However, the AUD prevalence we found among single participants was lower than that in the Nigeria study (35.4% vs. 66.7%, respectively).

In this study, male gender was associated with increased odds of all categories of alcohol use disorder, which is in agreement with previous study done in different countries [26, 28].

### Socio-economic factors associated with AUDs

In this study, earning annual income of 2,400-5,399 Ethiopian birr and annual income of 5,400-10,800 Ethiopian birr were significantly associated with alcohol use disorder (OR 0.22, 95% CI = 0.12,0.41 and OR 0.05, 95% CI = 0.02,0.10) compared to annual income of less than 2400 Ethiopian birr. This indicated that, as income of the individual increased the probability to have alcohol use disorder decreases.

### HIV-related factors and AUDs

In this study, the prevalence of AUDs in the ART-naïve group was 38.6% which was higher than the prevalence of AUDs among those who had taken ART (31.6%). The potential effect of pre-ART counseling offered by the ART clinic health professionals might explain the observed difference. The prevalence of AUDs among ART naïve patients (38.6%) was lower than the result from similar study done in Nigeria (57%) [26]. The prevalence of hazardous drinking among PLHIV who has taken ART was 24% which was higher than a similar study carried out in south west Uganda (15.4%) [27]. However, it was less than that found in a similar study from Nigeria (42%) [26].

AUD was higher among patients who did not take their ART medication as prescribed compared to patients who were taking their ART drugs appropriately (44% (n = 11) vs. 30.6% (n = 94)). It is possible that patients who missed taking doses of ART drugs so that they could continue to drink alcohol. It also indicates that when working to improve adherence to ART, it is important to consider AUD as one factor.

Participants categorized as being in WHO’s stage two of HIV/AIDS classification were more often identified to have higher AUDs than those who were categorized as being in stage four (40.6% vs. 21.1%). Since stage four of HIV/AIDS classification is a worse HIV stage, people who are in this stage may drink less as they are too ill. Similarly patients with CD4 ≥ 200 cells/mm^3^ were identified to have higher AUDs than patients with CD4 < 200 cells/mm^3^ (36.7% vs. 10.3%).

### Substance use, mental distress and AUDs

AUD prevalence among mentally distressed participants was higher than those who had low levels of mental distress (40.3% vs. 26.3%). It is possible that patients with mental distress use alcohol as a self-treatment, but AUDs are also associated with increased risk of developing mental distress. A cause-effect relationship could not be established because our study employed a cross-sectional study design.

### Limitations

This study used AUDIT to determine the presence of probable AUD. AUDIT is a screening tool and may not give an accurate estimate of alcohol use disorders. Furthermore, AUDIT was not validated in our population even though it has been shown to be useful in screening for AUDs cross-culturally. The absence of a validated cut-off point for persons living with HIV is a further limitation. Social desirability bias could be an important limitation as persons who use alcohol and other substances tend to under-report or deny their use when questionnaires are administered by interview. Although we went to extensive efforts to adapt the concept of a ‘standard drink’ to the Ethiopian setting, the absence of a policy defining the standard alcohol drink in Ethiopia was a limitation. Lastly, we relied upon self-report of adherence to ART which is likely to underestimate non-adherence.

## Conclusions

The high prevalence of hazardous and dependent alcohol use disorders found in this study of PLHIV in Ethiopia highlights a need to improve detection and treatment of alcohol use disorders as an integrated part of HIV services. Our study shows that AUDs go hand-in-hand with mental distress and use of other substances, which are likely to have cumulative effects on prognosis and quality of life in PLHIV.
